# Amelioration of Acetaminophen-Induced Hepatic Oxidative Stress and Inflammation by RNAi Targeting *Cyp2e1* In Vivo

**DOI:** 10.3390/cimb47050372

**Published:** 2025-05-19

**Authors:** Wenwen Liu, Liwen Huan, Cai Zhang, Runting Yin, Zhen Ouyang, Yuan Wei

**Affiliations:** School of Pharmacy, Jiangsu University, Zhenjiang 212013, China; wwen0239@163.com (W.L.); hlw102077@163.com (L.H.); caizhang@ujs.edu.cn (C.Z.); yinrunting@126.com (R.Y.); zhenouyang@ujs.edu.cn (Z.O.)

**Keywords:** acetaminophen, hepatotoxicity, *Cyp2e1*, RNAi, lipid nanoparticle, oxidative stress, inflammation, lipid metabolism

## Abstract

The overdose of acetaminophen (APAP) has become the leading cause of acute liver failure in the United States and some Western countries. As a principal member of the cytochrome P450 enzymes (CYPs), CYP2E1 is a vital enzyme in regard to the production of toxic APAP metabolites and in the development of APAP-induced liver injury (AILI). In this study, we investigated the therapeutic effects and mechanisms of lipid nanoparticle (LNP)-based delivery of small interfering RNA targeting *Cyp2e1* (si-*Cyp2e1* LNPs) on AILI in mice. C57BL/6J male mice were injected with 300 mg/kg APAP to establish an AILI model, and si-*Cyp2e1* LNPs were administered via the tail vein. The results showed that the levels of serum alanine aminotransferase and aspartate aminotransferase were lower than those in APAP mice after treatment with si-*Cyp2e1* LNPs immediately. Moreover, si-*Cyp2e1* LNPs significantly inhibited liver necrosis and oxidative stress in APAP mice. RNA sequencing revealed that si-*Cyp2e1* LNPs exerted regulatory effects on pathways and genes related to peroxisome proliferator-activated receptor (PPAR). Consistent with this finding, we also proved that si-*Cyp2e1* LNPs markedly regulated the expressions of genes involved in the PPAR signaling pathway (CYP4A, PPARα, FABP 1, and CD36) in APAP mice, as well as inflammatory factors (*Il-6*, *Il-1β*, and *Tnf-α*). These findings suggested that si-*Cyp2e1* LNPs may alleviate APAP-induced oxidative stress and inflammation by regulating lipid metabolism via PPAR-related pathways.

## 1. Introduction

Drug-induced liver injury (DILI), characterized by hepatic damage caused by drugs or their metabolites, is one of the most common and severe adverse reactions. Many drugs, including anticancer, anti-tuberculosis, and non-steroidal anti-inflammatory drugs (NSAIDs), can cause DILI to varying degrees [1,2]. Acute liver injury induced by excess acetaminophen (APAP; Supplementary Figure S1A), which is the most commonly used medication for pain and fever, is the leading cause of acute liver failure in the United States and the United Kingdom [3,4].

APAP-induced liver injury (AILI) is initiated by the conversion of N-acetyl-p-benzoquinone imine (NAPQI), which is primarily metabolized by cytochrome P450 enzymes (CYPs) [5]. High doses of APAP and NAPQI deplete glutathione (GSH). Excessive NAPQI can combine with mitochondrial proteins to form APAP protein adducts (APAP-ADs), thus triggering electron leakage, leading to the formation of mitochondrial superoxide, initiating oxidative stress and activating redox-sensitive mitogen-activated protein kinase (MAPK), which ultimately leads to the phosphorylation of c-jun N-terminal kinase (JNK) [6]. Moreover, peroxynitrite, generated by the rapid reaction of superoxide radicals with nitric oxide, further amplifies mitochondrial dysfunction and hepatocyte necrosis [7]. *N*-acetyl-*L*-cysteine (NAC; Supplementary Figure S1B) is the only clinically approved antidote for APAP overdose, which works by restoring GSH levels in the early phase [8]. However, the mortality rate of the overall cases of APAP overdose has changed slightly over the past 20 years, despite the presence of NAC [9]. Therefore, novel alternatives to NAC or supplements for treating APAP overdose need to be developed.

CYP2E1 has been confirmed as a vital enzyme responsive to NAPQI generation and to the development of AILI [10]. Enhanced sensitivity to APAP hepatotoxicity and oxidative stress is observed after CYP2E1 induction [11,12]. Oxidative stress and liver injury are markedly ameliorated after the inhibition or knockout of CYP2E1 in many hepatic diseases [13,14,15]. Moreover, 4-methylpyrazole, an inhibitor of CYP2E1, is already undergoing clinical trials as a supplement to NAC for the treatment of APAP overdose [9], demonstrating the potential of drugs targeting CYP2E1 inhibition.

RNA interference (RNAi) is a new clinical therapy used to treat diseases by silencing specific target genes and blocking protein expression in vivo. However, it is difficult for small interfering RNA (siRNA) to enter cells because of their large molecular size, strong hydrophilicity, and anionic charge. Therefore, different drug carriers are required for delivery [16]. Lipid nanoparticle (LNP) delivery systems are characterized by high safety, low immunogenicity, and strong targeting abilities. Our previous research showed that an LNP-delivered siRNA targeting *Cyp2e1* (si-*Cyp2e1* LNPs) could effectively inhibit the development of chronic and subacute alcoholic liver disease in mouse models [17,18]. However, the effect of si-*Cyp2e1* LNPs in acute liver injury induced by APAP overdose in mice has not yet been investigated.

In this study, the therapeutic potential of si-*Cyp2e1* LNPs in a mouse model of AILI was demonstrated, and the underlying mechanisms were explored through transcriptomics.

## 2. Materials and Methods

### 2.1. Chemicals

APAP and NAC were purchased from Yuanye Biotechnology (Shanghai, China). (6Z, 9Z, 28Z, 31Z)-heptatriacont-6,9,28,31-tetraene-19-yl 4-(dimethylamino) butanoate (Dlin-MC3-DMA) and cholesterol were purchased from AVT Pharmaceutical Tech Co., Ltd. (Shanghai, China). Distearoyl phosphatidylcholine (DSPC) was purchased from Macklin Biochemical Technology (Shanghai, China). 1,2-dimyristoyl-sn-glycero-3-phosphoethanolamine-*N*-[methoxy(polyethylene glycol)-2000] (C14-PEG2000) was obtained from Sigma Aldrich (St. Louis, MO, USA).

### 2.2. Preparation and Characterization of LNPs

As previously described, an siRNA targeting mouse *Cyp2e1* was designed (Supplementary Table S1) [17]. Single-stranded RNAs were synthesized and then annealed into double-stranded siRNAs by GenScript (Nanjing, China). LNPs were prepared as previously described [19]. Briefly, siRNAs were dissolved in 10 mmol/L sodium acetate buffer, and Dlin-MC3-DMA, cholesterol, DSPC, and C14-PEG2000 were dissolved and mixed in ethanol at a molar ratio of 50/38.5/10/1.5, respectively. Then, the lipid and siRNAs were mixed at a volume and velocity ratio of 1/3 through a microfluidic device (FluidicLab, Shanghai, China). Lastly, the mixture was dialyzed against Dulbecco’s phosphate-buffered saline (DPBS; pH 7.4) for 2 h using a 20,000 molecular weight cut-off (MWCO) dialysis bag at 25 °C. The LNPs were characterized using a NanoBrook 90Plus PALS system (Brookhaven, Holtsville, NY, USA), and the encapsulation efficiency was measured according to established procedures [20]. Morphology was observed by transmission electron microscopy (TEM) (Hitachi, Tokyo, Japan).

### 2.3. Animal Experiments

Healthy 6–8-week-old male C57BL/6J mice (18–22 g) were sourced from and housed in the Laboratory Animal Research Center of Jiangsu University (license nos. SCXK (Su) 2023-0017 and SYXK (Su) 2023-0081). Experimental mice were housed in a standard environment, with a room temperature of 20–24 °C, relative humidity of 50–60%, and 12 h light/dark cycles. All animal experiments were approved by the Institutional Animal Care and Use Committee of Jiangsu University (approval number: UJS-IACUC-2023122802).

In this study, si-C*yp2e1* was generated by *Cyp2e1* knockdown using RNAi. The mice were injected with 0.5 mg/kg si-*Cyp2e1* LNPs or si-Control LNPs dissolved in DPBS via the tail vein to confirm their silencing effect [18]. The mice injected with the same dose of DPBS served as a negative control group. Each group consisted of four mice, and each mouse received a single injection. The mice were euthanized via intraperitoneal administration of sodium pentobarbital 24 h after tail vein injection [17]; then, the livers were harvested. Subsequently, *Cyp2e1* expression in the liver was assessed.

The AILI mouse model used in this study was modified from a previous report [21]. The experimental mice were randomly divided into five different groups, including the control group (Ctrl), APAP group (APAP), APAP + si-*Cyp2e1* LNPs group (si-*Cyp2e1* LNPs), APAP + si-Control LNPs group (si-Control LNPs), and APAP + NAC group (NAC) (*n* = 4). All the experimental animals were fasted for 12 h before drug administration. In addition to the Ctrl group, the mice in the other four groups received intraperitoneal injections of 300 mg/kg APAP dissolved in hot saline. The mice in the Ctrl group were administered the same volume of normal saline. Subsequently, the mice in the si-*Cyp2e1* and si-Control treatment groups were immediately injected with 0.5 mg/kg LNPs through the tail vein. The mice in the positive drug treatment group received immediate intragastric administration of NAC at a dose of 200 mg/kg. A total of 24 h after model establishment, all the mice were anesthetized by intraperitoneal injection of pentobarbital sodium and subsequently euthanized after blood collection. Complete fresh liver tissues were collected, one lobe of liver tissue was soaked in 4% paraformaldehyde fixing solution, and the remaining liver tissues were stored in a refrigerator at −80 °C for further use.

To investigate the effect of delayed treatment, all the mice were divided into five groups: Ctrl, APAP, and three treatment groups (*n* = 4). The administration of 300 mg/kg APAP and normal saline was consistent with the experiment above. In contrast to the previous, 0.5 mg/kg LNPs or 200 mg/kg NAC was administered once, 2 h after APAP overdose. After 24 h of modeling, blood and fresh liver samples were collected, and one lobe of the liver was soaked in paraformaldehyde.

A previous study demonstrated that a 2 h delayed treatment with 100 mg/kg NAC every 12 h for three consecutive days delayed the recovery of AILI mice in the late stage [22]. To determine whether delayed treatment with si-*Cyp2e1* LNPs affects liver recovery of AILI mice, the modeling period was extended to 72 h. All the mice were divided into four groups: Ctrl, APAP, si-*Cyp2e1* LNPs delayed treatment, and NAC delayed treatment (*n* = 4). The administration of 300 mg/kg APAP and saline was consistent with the above experiments, and the mice were treated 2 h after APAP overdose. The si-*Cyp2e1* LNPs-treated mice received a single tail vein injection of 0.5 mg/kg LNPs, which was previously shown to have a significant gene silencing effect for 72 h [23]. The NAC treatment group received 100 mg/kg NAC by oral gavage every 12 h. A total of 72 h after APAP injection, the livers were collected for subsequent pathological analysis.

### 2.4. Serum and Liver Analysis

The liver index was calculated as the ratio of liver weight to body weight. Serum was isolated by centrifuging whole blood, and the homogenate was obtained by mixing and grinding the tissue with a buffer solution. Levels of serum alanine aminotransferase (ALT), aspartate aminotransferase (AST), hepatic glutathione (GSH), malondialdehyde (MDA), and superoxide dismutase (SOD) were determined according to the instruction of kits purchased from Nanjing Jiancheng Bioengineering Institute (Nanjing, China). The levels of oxidative stress indicators in the liver were normalized to the corresponding tissue protein content, which was measured using the BCA Protein Assay Kit (GlpBio, Montclair, CA, USA).

In order to determine reactive oxygen species (ROS) levels, fresh liver tissue was homogenized on ice for 30 s after adding the buffer. The samples were centrifuged once (100× *g*, 4 °C, 10 min), and the supernatant was removed. According to the instructions of the ROS activity detection kit (BestBio Technology, Shanghai, China), the supernatant was mixed with the O06 probe and incubated at 37 °C for 30 min, and the fluorescence intensity of the product was detected using a fluorescent enzyme spectrometer (excitation wavelength 488 nm, emission wavelength 526 nm). The corresponding tissue protein concentration was determined, and the ratio of fluorescence intensity to tissue protein content was used as the level of reactive oxygen species in the tissue for data analysis.

### 2.5. Liver Pathology

The liver tissue was fixed in 4% paraformaldehyde, embedded in paraffin, and sectioned (4 μm). After dewaxing and dehydration, the slices were stained with hematoxylin and eosin (H&E; Servicebio, Wuhan, China) for 4 min and 15 s, respectively. Finally, the sections were sealed with neutral gum after dehydration. Representative images were captured using a pathological slide scanner (3DHISTECH, Budapest, Hungary).

### 2.6. RNA Sequencing and Enrichment Analysis

Total RNA was extracted using TRIzol (Thermo Fisher Scientific, Waltham, MA, USA), and the concentration was determined. RNA enrichment and sequencing library construction were performed using mRNA capture and fast RNA library kits (Tiangen Biotech, Beijing, China). Qualified libraries were sequenced on a NovaSeq6000 platform (Illumina, San Diego, CA, USA). The raw data were filtered to obtain high-quality clean reads, which were mapped to the reference genome using HISAT2 [24]. Gene expression quantification was performed using StringTie [25]. Limma was used to screen differentially expressed genes (DEGs) in the APAP and Ctrl groups, as well as in the si-*Cyp2e1* LNPs-treated and APAP groups [26]. The DEGs were screened based on the criteria *p* < 0.05 and log_2_|Fold Change| > 0.585. Finally, enrichment analysis of DEGs was performed using Gene Ontology (GO), Kyoto Encyclopedia of Genes and Genomes (KEGG), and gene set enrichment analysis (GSEA). The enrichplot package in R v4.4.2 was used for visualization, with a Q value < 0.05 as the screening criterion.

### 2.7. Quantitative Real-Time Polymerase Chain Reaction (qPCR)

RNA reverse transcription and qPCR were performed according to a previous study [17]. Each program ran 45 cycles and was carried out three times. Gene expression levels were calculated according to the 2^−ΔΔCt^ method, and glyceraldehyde-3-phosphate dehydrogenase (*Gapdh*) was used as an internal reference. The PCR primers (Supplementary Table S2) were synthesized by Sangon Biotech (Shanghai, China).

### 2.8. Western Blotting

The mouse livers were lysed by RIPA buffer to obtain total proteins. The proteins were quantified using a BCA Protein Assay Kit (GK10009, GlpBio). The extracted proteins and loading buffer (5×) were mixed and dissolved in a ratio of 4:1. The proteins (20 μg) were resolved by SDS-PAGE and transferred to a PVDF membrane, which was closed for 1.5 h using 5% skim milk powder at room temperature. Next, the membrane was separately probed with the primary antibodies CYP2E1 (67263-1-Ig, Proteintech, Rosemont, Il, USA, 1:1000), CYP4A (sc-374537, Santa Cruz, Dallas, TX, USA, 1:1000), CD36 (sc-7309, Santa Cruz, 1:1000), L-FABP (sc-374537, Santa Cruz, 1:1000), and GAPDH (10494-1-AP, Proteintech, 1:1000) overnight at 4 °C, and the non-specifically bound proteins were washed off. Subsequently, the bands were incubated with the corresponding secondary antibodies (Beyotime Biotechnology, Shanghai, China, 1:3000) for 1 h. After washing off the unbound proteins, the membrane was visualized using an enhanced chemiluminescence kit (GK10008, GlpBio) and a ChemiDoc XRS imaging system (Bio-Rad, Hercules, CA, USA). Gray values were analyzed using ImageJ v1.51, and GAPDH was used as an internal reference to calculate the relative expression of the target proteins.

### 2.9. Statistical Analysis

The data are presented as means ± standard deviations (SDs) and were analyzed using one-way analysis of variance (ANOVA) in GraphPad Prism 8.3.0. A *p*-value < 0.05 was considered to indicate a statistically significant difference. All experiments were performed in triplicate.

## 3. Results

### 3.1. Characterization and Validation of si-Cyp2e1 LNPs In Vivo

**Figure 1:**
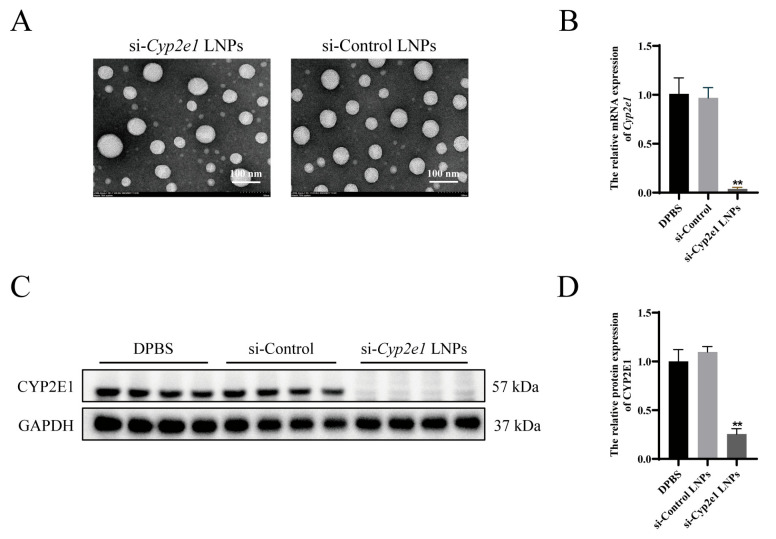
Characterization of si-*Cyp2e1* LNPs and knockdown efficiency in vivo (*n* = 4). (**A**) Representative image under transmission electron microscopy (scale = 100 nm). (**B**) *Cyp2e1* mRNA expression in the liver 24 h after a single injection of si-*Cyp2e1* LNPs. (**C**,**D**) CYP2E1 protein expression and quantification. ** *p* < 0.01 vs. the DPBS group.

The characterization results are presented in Table 1. The TEM images revealed that the si-*Cyp2e1* LNPs exhibited a spherical morphology with a particle size of approximately 80 nm, which can be used to target the liver (Figure 1A).

The hepatic expression levels of *Cyp2e1* were quantitatively assessed 24 h after a single tail vein injection. qPCR analysis showed that *Cyp2e1* mRNA expression was reduced by over 90% in the mice injected with si-*Cyp2e1* LNPs compared to that in the mice receiving DPBS (Figure 1B). Western blotting also indicated that the CYP2E1 protein level decreased by approximately 70% 24 h after tail vein injection of si-*Cyp2e1* LNPs (Figure 1C,D).

### 3.2. Immediate Treatment with si-Cyp2e1 LNPs Could Alleviate AILI in Mice

**Figure 2:**
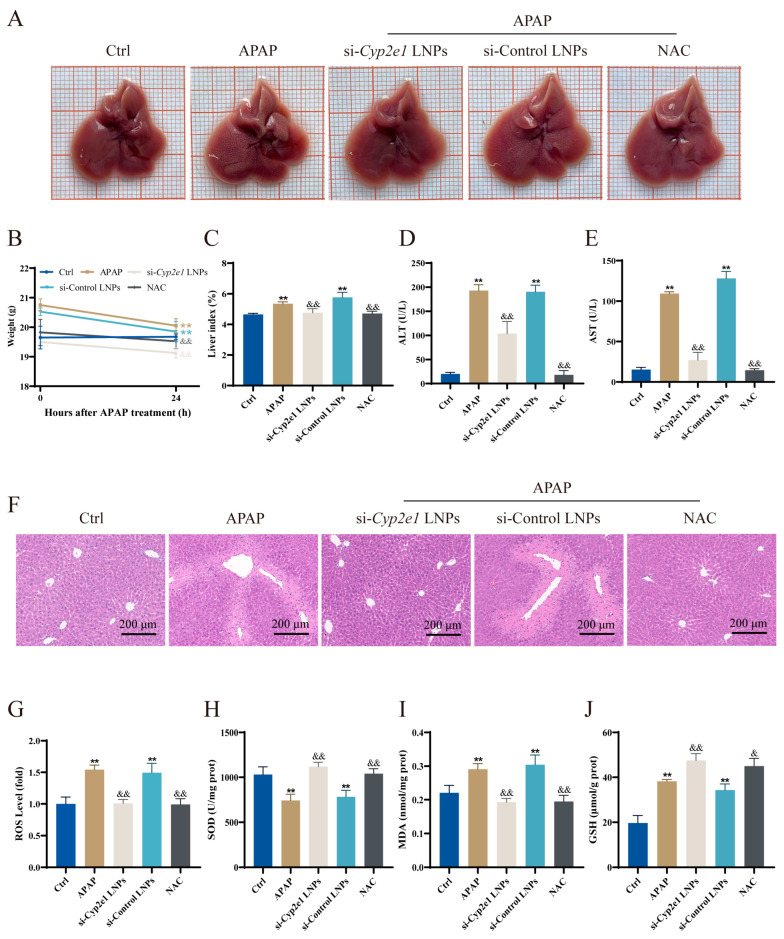
Effects of immediate treatment with si-*Cyp2e1* LNPs on APAP-injected mice (*n* = 4). (**A**) Appearance morphology of mouse livers. (**B**) Changes in the body weight of the mice. (**C**–**E**) Levels of liver index, serum ALT, and AST. (**F**) H&E staining of mouse livers (scale bars = 200 μm). (**G**–**J**) Levels of ROS, SOD, MDA, and GSH in mouse livers 24 h after a single dose of 300 mg/kg APAP. ** *p* < 0.01 vs. the Ctrl group; & *p* < 0.05 and && *p* < 0.01 vs. the APAP group.

All of the mice survived throughout the whole experiment. The liver appearance of the mice was observed after treatment with different drugs immediately after APAP administration; NAC served as a positive control. The surface texture of the liver in the APAP and si-Control LNPs groups was slightly rough with an obvious granular appearance (Figure 2A). However, the liver surfaces of the mice treated with si-*Cyp2e1* LNPs or NAC were smooth, without a granular appearance, and showed no evident difference from the Ctrl group (Figure 2A). When compared to the Ctrl group, the mice injected with APAP showed a decrease in body weight at 24 h (Figure 2B). The results of mouse body weight and liver index analysis indicated that the livers of the APAP mice showed significant hepatomegaly compared to those of the Ctrl mice, and the livers returned to normal size after treatment with si-*Cyp2e1* LNPs or NAC immediately (Figure 2B,C).

The serum levels of ALT and AST were used as sensitive indicators of liver injury [27,28]. When compared to the Ctrl group, these indices sharply increased in the APAP group, indicating that the DILI mouse model was successfully established. However, si-*Cyp2e1* LNPs significantly reversed this increase in the APAP mice (Figure 2D,E). Consistent with these findings, H&E staining revealed disordered hepatic lobules in the APAP mice, with numerous necrotic hepatocytes around the portal area and inflammatory infiltration. In contrast, edema and inflammation were considerably alleviated in the si-*Cyp2e1* LNPs and NAC groups, and the hepatic lobules were clearly organized and closely arranged (Figure 2F). In summary, the progression of AILI was slowed by si-*Cyp2e1* LNPs.

To elucidate the effects of si-*Cyp2e1* LNPs on the oxidative status, we evaluated the levels of ROS, GSH, SOD, and MDA in the liver. Excessive ROS accumulation triggers oxidative stress and mitochondrial dysfunction [29]. Elevated hepatic MDA levels are indicative of increased lipid peroxidation, potentially culminating in hepatocellular injury and diminished antioxidant defenses [30]. Hepatic ROS and MDA levels significantly increased 24 h after APAP injection, whereas si-*Cyp2e1* LNPs significantly inhibited this increase and oxidation effect (Figure 2G,H). In contrast to ROS and MDA, the levels of SOD and GSH reflect the antioxidant capacity of the body. The results showed that the liver SOD content was significantly reduced after APAP treatment compared to that in the Ctrl group, while si-*Cyp2e1* LNPs restored the antioxidant capacity of the APAP mice (Figure 2I). The GSH levels in the APAP mice recovered to levels higher than those in the Ctrl group 24 h after intraperitoneal injection of APAP. Interestingly, the hepatic GSH levels in the si-*Cyp2e1* LNPs and NAC groups were higher than those in the APAP group (Figure 2J).

### 3.3. Transcriptomics Revealed the Possible Mechanism by Which si-Cyp2e1 LNPs Attenuate AILI in Mice

The above results indicated that timely treatment with si-*Cyp2e1* LNPs could alleviate AILI in mice. Next, the mouse liver samples were sequenced to further explore the potential mechanism.

**Figure 3:**
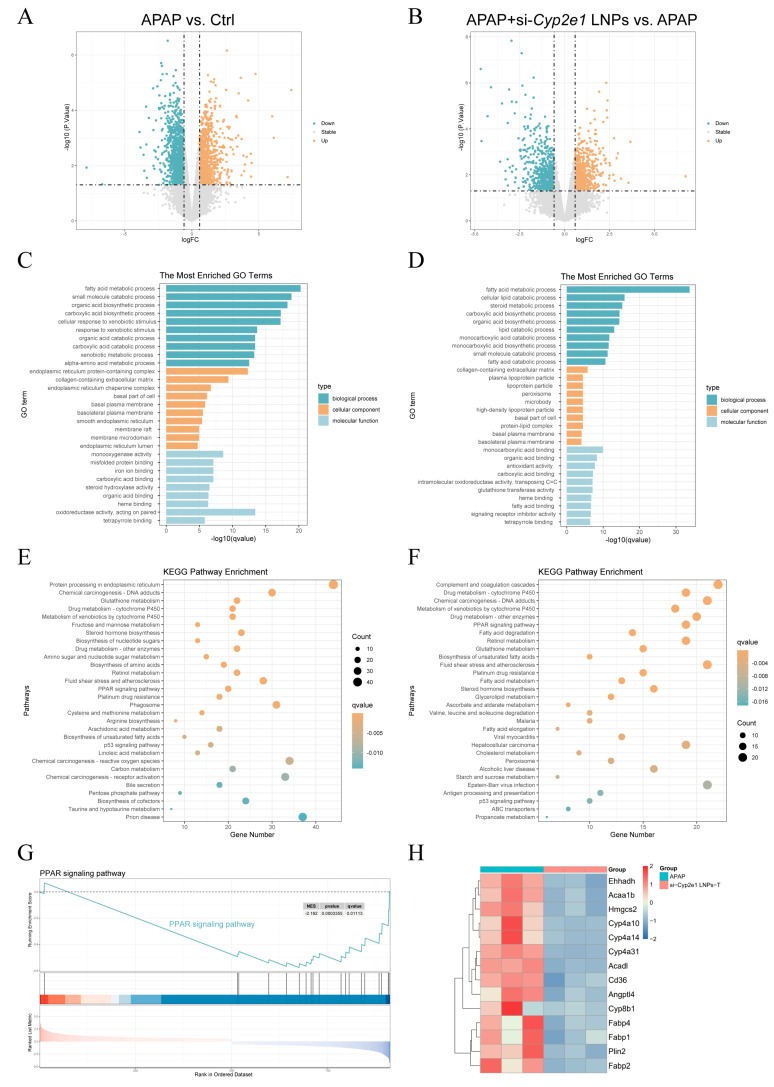
Transcriptomic analysis of si-*Cyp2e1* LNPs on AILI mice (*n* = 3). (**A**,**B**) Volcano map of the APAP group vs. the Ctrl group and the si-*Cyp2e1* LNPs treatment group vs. the APAP group. (**C**,**D**) GO analysis chart. (**E**,**F**) KEGG analysis. (**G**) GSEA of the PPAR signaling pathway between the si-*Cyp2e1* LNPs treatment and APAP groups. (**H**) Heatmap of the PPAR signaling pathways related gene expression.

The DEGs between the groups were compared and are presented (Supplementary Figure S2A,B). In contrast to the Ctrl group, there were 1053 upregulated genes and 1217 downregulated genes in the APAP group. A total of 1242 DEGs with 583 downregulated and 659 upregulated genes were present between the si-*Cyp2e1* LNPs and APAP groups (Supplementary Figure S2C and Figure 3A,B). The GO and KEGG analyses showed that APAP exerted regulatory effects on several physiological processes and signaling pathways. These included reactive oxygen species, protein processing in the endoplasmic reticulum (ER), misfolded protein binding, oxidoreductase activity, and the p53 and peroxisome proliferator-activated receptor (PPAR) signaling pathways. In the meantime, si-*Cyp2e1* LNPs affected cytochrome P450, fatty acid degradation and metabolism, unsaturated fatty acid biosynthesis, glutathione metabolism, cholesterol metabolism, and the PPAR signaling pathway (Figure 3C–F). From the results of GSEA, we found that the production of cytokines and tumor necrosis factor (TNF), glutathione metabolism, the ERK1 and ERK2 cascade, the MAPK cascade, and the regulation of the apoptotic signaling pathway in the APAP model group were notably upregulated compared to those in the Ctrl group (Supplementary Figure S3A). After si-*Cyp2e1* LNPs treatment, fatty acid degradation, unsaturated fatty acid biosynthesis, and the PPAR signaling pathway were significantly inhibited compared with those in the APAP group (Supplementary Figure S3B and Figure 3G). The heatmap further revealed a significant downregulation of genes associated with the PPAR signaling pathway (Figure 3H).

### 3.4. si-Cyp2e1 LNPs Could Regulate the PPAR Signaling Pathway

**Figure 4:**
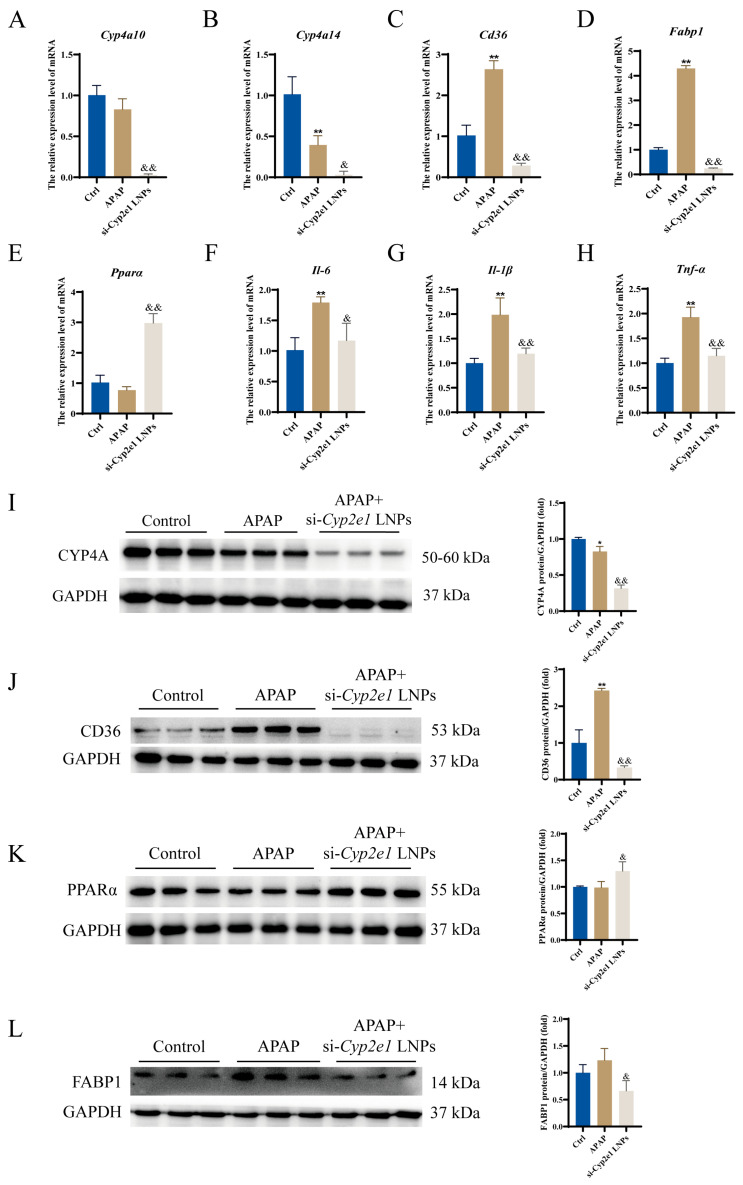
Effects of si-*Cyp2e1* LNPs on the genes involved in the PPAR signaling pathway and inflammation in the livers of APAP mice (*n* = 3). (**A**–**H**) mRNA expression of *Cyp4a10*, *Cyp4a14*, *Pparα*, *Cd36*, *Fabp1*, *Il-6*, *Il-1β*, and *Tnf-α*. (**I**–**L**) Protein levels of CYP4A, PPARα, CD36, and FABP1. * *p* < 0.05, ** *p* < 0.01 vs. the Ctrl group; & *p* < 0.05 and && *p* < 0.01 vs. the APAP group.

To further validate the findings of RNA sequencing, we verified the mRNA and protein levels separately. Similar to the previous results, the qPCR analysis indicated that the mRNA expression levels of *Cyp4a10*, *Cyp4a14*, fatty acid binding protein 1 (*Fabp1*), and cluster of differentiation 36 (*Cd36*) were significantly decreased in the si-*Cyp2e1* LNPs treatment group compared to those in the APAP group (Figure 4A–D). Notably, si-*Cyp2e1* LNPs upregulated the mRNA of *Pparα* (Figure 4E). Furthermore, inflammatory cytokines were significantly inhibited in the si-*Cyp2e1* LNPs-treated mice compared with the APAP mice, including interleukin (*Il)-6*, *Il-1β*, and *Tnf-α* (Figure 4F–H). Western blotting further demonstrated that si-*Cyp2e1* LNPs markedly downregulated CYP4A, CD36, and FABP1 proteins, while the PPARα levels were significantly increased compared with the APAP group (Figure 4I–L). These results suggest that si-*Cyp2e1* LNPs could regulate the genes involved in the PPAR signaling pathway and inflammation.

### 3.5. Delayed Treatment with si-Cyp2e1 LNPs for 2 h Did Not Ameliorate AILI in Time

We further investigated whether delayed treatment with si-*Cyp2e1* LNPs protected the mice from APAP hepatotoxicity. We injected si-*Cyp2e1* LNPs into the tail veins of the mice 2 h after APAP overdose. The therapeutic effect after 24 h of modeling is shown in Supplementary Figure S4. As shown in the mouse liver appearance and liver index, a 2 h delay in the si-*Cyp2e1* LNPs treatment did not alter the granular appearance or hepatomegaly caused by APAP (Supplementary Figure S4A–C). In addition, the serum ALT and AST levels did not exhibit a statistically significant difference between the APAP and si-*Cyp2e1* LNPs delayed treatment groups (Supplementary Figure S4D,E). Consistent with these findings, H&E staining indicated that delayed treatment with si-*Cyp2e1* LNPs did not improve local liver necrosis (Supplementary Figure S4F).

### 3.6. si-Cyp2e1 LNPs Might Accelerate Liver Recovery Compared with NAC

In both cellular and mouse models, previous reports have shown that delayed and continuous treatment with NAC may inhibit hepatocyte regeneration, affecting the recovery of liver function in the late stage of AILI [9,31]. To investigate the effect of delayed treatment with a single dose of 0.5 mg/kg si-*Cyp2e1* LNPs on liver recovery in the AILI mice, pathological analysis of the liver was performed 72 h after modeling. As shown in Supplementary Figure S5, significant inflammatory infiltration was still observed in the liver tissue of the APAP group when compared to that in the Ctrl group, as indicated by the black arrow. Continuous administration of NAC did not improve this inflammation, and areas of hepatocyte edema were observed, as indicated by the red arrow. Notably, si-*Cyp2e1* LNPs significantly ameliorated the hepatic pathological changes induced by APAP, and there were no significant differences between the si-*Cyp2e1* LNPs delayed treatment group and the Ctrl group, implying that si-*Cyp2e1* LNPs may accelerate liver recovery in the late phase of AILI in mice.

## 4. Discussion

Previous studies have demonstrated that excessive APAP is metabolized by CYPs into bioactive metabolites, leading to GSH depletion and ROS formation, triggering oxidative stress, mitochondrial permeability transition, and ATP loss, eventually resulting in hepatocyte necrosis [10]. During the development of AILI, CYP2E1 is a key enzyme in the generation of the hepatotoxic metabolite NAPQI, and the hepatotoxicity of APAP is enhanced after CYP2E1 induction [32]. In the present study, siRNA-LNPs targeting *Cyp2e1* were used to investigate their therapeutic effects against AILI in mice. The measured particle size of si-*Cyp2e1* LNPs was approximately 80 nm, demonstrating effective hepatic targeting [33]. In subsequent pharmacodynamic experiments, the levels of liver injury and oxidative stress-related indicators significantly changed 24 h after APAP administration. Fortunately, all indices, except GSH, tended to be normal after immediate treatment with si-*Cyp2e1* LNPs, and the area of liver tissue necrosis was markedly reduced. According to previous reports, GSH is depleted 2 h after APAP overdose, gradually recovers from 6 h, and returns to above-normal levels by 24 h [34,35]. This phenomenon of body self-replenishment of GSH was reflected in our experimental results and was more significant in the mice treated with si-*Cyp2e1* LNPs. These results implied that targeted inhibition of *Cyp2e1* could effectively alleviate oxidative stress and AILI in mice.

RNA sequencing and enrichment analysis showed that APAP could induce significant changes in protein processing in the ER, misfolded protein binding, ROS, production of cytokine and TNF, ERK and MAPK cascades, apoptosis, and the p53 signaling pathway, indicating that APAP could induce ER stress, oxidative stress, the inflammatory response, and hepatocyte apoptosis. In the development of APAP hepatotoxicity, initial ROS formation leads to the activation of MAPK and the phosphorylation of JNK, followed by p-JNK translocation to the mitochondria, which results in further amplification of mitochondrial ROS release [36]. Subsequently, the p53 signaling pathway is activated in response to cellular stress and injury [6]. These events are consistent with our findings. We found that the PPAR signaling pathway might be an important pathway for si-*Cyp2e1* LNPs to alleviate AILI in mice, as verified by subsequent research.

Similar to CYP2E1, the CYP4A family comprises microsomal oxidases involved in fatty acid oxidation, both of which can reduce molecular oxygen to produce prooxidants, leading to oxidative stress if antioxidants are ineffective. Mouse CYP4A10 and CYP4A14 are important components of CYP4A, which not only catalyze the ω-hydroxylation of fatty acids [37] but also activate the expression of inflammatory factors through the ROS/NF-κΒ pathway [38]. Studies have shown that CYP2E1 can be induced by long-term alcohol consumption or a high-fat diet, while the knockout or continuous inhibition of *Cyp2e1* leads to a compensatory increase in CYP4A [39,40]. Our previous research showed that the simultaneous inhibition of *Cyp2e1*, *Cyp4a10*, and *Cyp4a14* significantly inhibited oxidative stress in mice with alcoholic liver injury [41]. Interestingly, the present study indicated that short-term targeted inhibition of CYP2E1 in vivo resulted in significant suppression of CYP4A expression in mice exposed to APAP. Under such a condition, the alleviation of APAP-induced oxidative stress by si-*Cyp2e1* LNPs may be attributed to the effective inhibition of CYP2E1 and CYP4A. These observations provided new insights into the interactions between CYPs under varying circumstances in vivo.

In the present study, we found that si-*Cyp2e1* LNPs not only downregulated the expressions of CD36, FABP1, and inflammatory factors but also increased the level of PPARα in the liver. CD36 is a scavenger receptor that mediates lipid intake, the adhesion of immune recognition cells, inflammation, and apoptosis [42], and it is a key factor involved in fat degeneration and liver damage [43]. Studies have proved that CYP2E1 inhibitors would decrease CD36 expression in vivo and in vitro [44] and that CD36 deficiency attenuates AILI in mice by inhibiting JNK activation and HMGB1-mediated inflammatory responses [45]. PPARs are critical for fatty acid catabolism, and PPARα is a key molecule that controls lipid synthesis and fatty acid oxidation pathways [46]. PPARα is activated in *Cyp2e1* knockout mice, which is attributed to the accumulation of its endogenous substrates, identified as direct PPARα agonists [47,48]. A PPARα activator attenuated APAP hepatotoxicity in wild-type mice, whereas this protective effect was absent in *Pparα*^−/−^ mice [49,50]. FABP1 is downstream of the PPAR signaling pathway, and the silencing of FABP1 in the liver ameliorated hepatic oxidative stress and inflammation in a mouse model of nonalcoholic fatty liver disease [51]. Lipid peroxidation is often considered an important mechanism by which ROS regulate hepatocyte death induced by APAP overdose [52]. ERK, JNK, and p38 form adducts with the products of lipid peroxidation to activate the MAPK signaling pathway [53]. We found that si-*Cyp2e1* LNPs regulated lipid metabolism and inhibited lipid peroxidation by activating PPARα while inhibiting unsaturated fatty acid biosynthesis and fatty acid degradation. These results suggest that si-*Cyp2e1* LNPs may protect the mouse liver from inflammation and injury triggered by APAP through the regulation of PPAR-related pathways. However, the effects of si-*Cyp2e1* LNPs on other pathways, such as the JNK, MAPK, and p38 signaling pathways, and their interactions remain to be studied.

Next, we explored the effect of a 2 h delayed treatment with si-*Cyp2e1* LNPs. Unfortunately, the results of delayed treatment did not meet our expectations, probably due to the rapid metabolism of APAP in vivo. As previously mentioned, GSH levels recovered 6 h after APAP overdose [34], whereas our recent study showed that si-*Cyp2e1* LNPs required 6 h after injection to significantly inhibit CYP2E1 expression [23], implying that delayed treatment for 2 h might not inhibit the formation of NAPQI and APAP adducts. Therefore, delayed si-*Cyp2e1* LNPs treatment did not have the same therapeutic effect as immediate administration during the early phase of AILI. Interestingly, we observed that si-*Cyp2e1* LNPs could accelerate hepatic recovery in the late stages of AILI. However, this conclusion is preliminary, and changes in the biomarkers of liver regeneration remain to be further determined.

As the only antidote for APAP overdose in clinical practice, NAC still has many drawbacks, such as delayed liver recovery after prolonged treatment and adverse gastrointestinal reactions [22]. Our present study has demonstrated that LNP-delivered siRNA targeting *Cyp2e1* could alleviate acute liver injury induced by APAP overdose, providing a new therapeutic strategy for rescuing APAP poisoning and broadening the application of RNAi in acute liver disease. In the future, RNAi therapy could be considered for the treatment of other drug metabolism-related diseases.

## 5. Conclusions

In summary, our study demonstrated that RNAi targeting *Cyp2e1* in vivo could not only alleviate APAP-induced oxidative stress and inflammation in mice by regulating lipid metabolism through PPAR-related pathways, but it could also contribute to liver recovery in the later stages of the injury, illustrating the therapeutic potential of RNAi therapy in acute liver disease and hepatic metabolism-related diseases in the future.

## Figures and Tables

**Table 1:** Characterization of lipid nanoparticles (LNPs).

Name	Size (nm)	Polydispersity Index	Zeta Potential (mV)	Encapsulation Rate (%)
si-*Cyp2e1* LNPs	78.04 ± 0.29	0.143 ± 0.031	−0.45 ± 0.1	89.05 ± 0.38
si-Control LNPs	72.47 ± 2.00	0.144 ± 0.013	−0.37 ± 0.1	85.86 ± 1.07

## Data Availability

The raw sequencing data were deposited in the NCBI under the BioProject number PRJNA1211279. The data will be made available on request.

## Supplementary Materials

The following supporting information can be downloaded at: https://www.mdpi.com/article/10.3390/cimb47050372/s1.

## Abbreviations

APAP	acetaminophen
AILI	APAP-induced liver injury
NAPQI	*N*-acetyl-*p*-benzoquinone imine
CYPs	cytochrome P450 enzymes
RNAi	RNA interference
siRNA	small interfering RNA
LNPs	lipid nanoparticles
ALT	alanine aminotransferase
AST	aspartate aminotransferase
ROS	reactive oxygen species
MDA	malondialdehyde
SOD	superoxide dismutase
GSH	glutathione
H&E	hematoxylin and eosin
DEGs	differentially expressed genes
GO	Gene ontology
KEGG	Kyoto encyclopedia of genes and genomes
GSEA	gene set enrichment analysis
qPCR	quantitative real-time polymerase chain reaction
GAPDH	glyceraldehyde-3-phosphate dehydrogenase
PPAR	peroxisome proliferator-activated receptor
CD36	cluster of differentiation 36
FABP1	fatty acid binding protein 1
IL	interleukin
TNF	tumor necrosis factor
