# Supplementary material for: Amelioration of Acetaminophen-Induced Hepatic Oxidative Stress and Inflammation by RNAi Targeting Cyp2e1 In Vivo
Source: Curr Issues Mol Biol. 2025 May 19;47(5):372. doi: 10.3390/cimb47050372 (PMC12110742; doi:10.3390/cimb47050372)
Supplement: Supplementary file 1 [file cimb-47-00372-s001.zip › cimb-3622004-supplementary/Supplementary Figures/Supplementary Figures.pdf]

Amelioration of Acetaminophen-Induced Hepatic Oxidative Stress and Inflammation by RNAi Targeting *Cyp2e1 in vivo*

Wenwen Liu, Liwen Huan, Cai Zhang, Runting Yin, Zhen Ouyang and Yuan Wei *

School of Pharmacy, Jiangsu University, Zhenjiang 212013, Jiangsu, China; wwen0239@163.com (W.L.)

* Correspondence: ywei@ujs.edu.cn

Supplementary Figures

A B

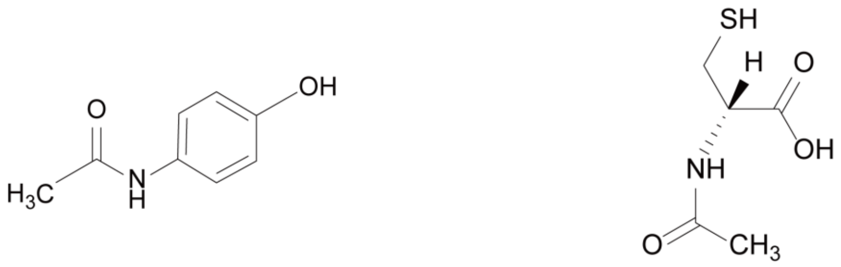

Figure S1. Chemical structures. **(A,B)** Structure of acetaminophen and *N*-acetyl-*L*-cysteine.

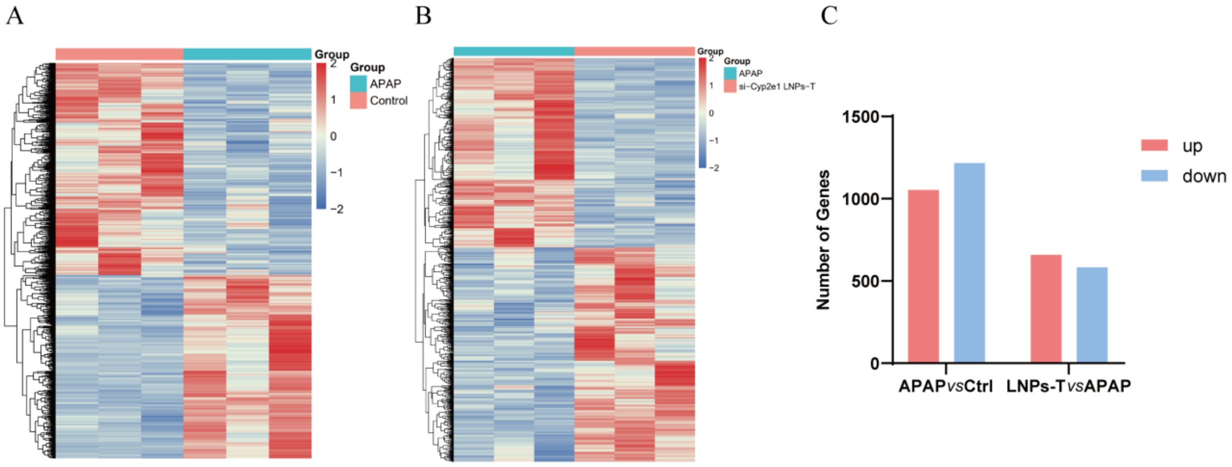

Figure S2. Data preprocessing for transcriptome data analysis. **(A,B)** Analysis of significantly differentially expressed genes (DEGs) between APAP and Ctrl groups, as well as si-*Cyp2e1* LNPs treatment and APAP groups. **(C)** Quantitative analysis of DEGs in different groups.

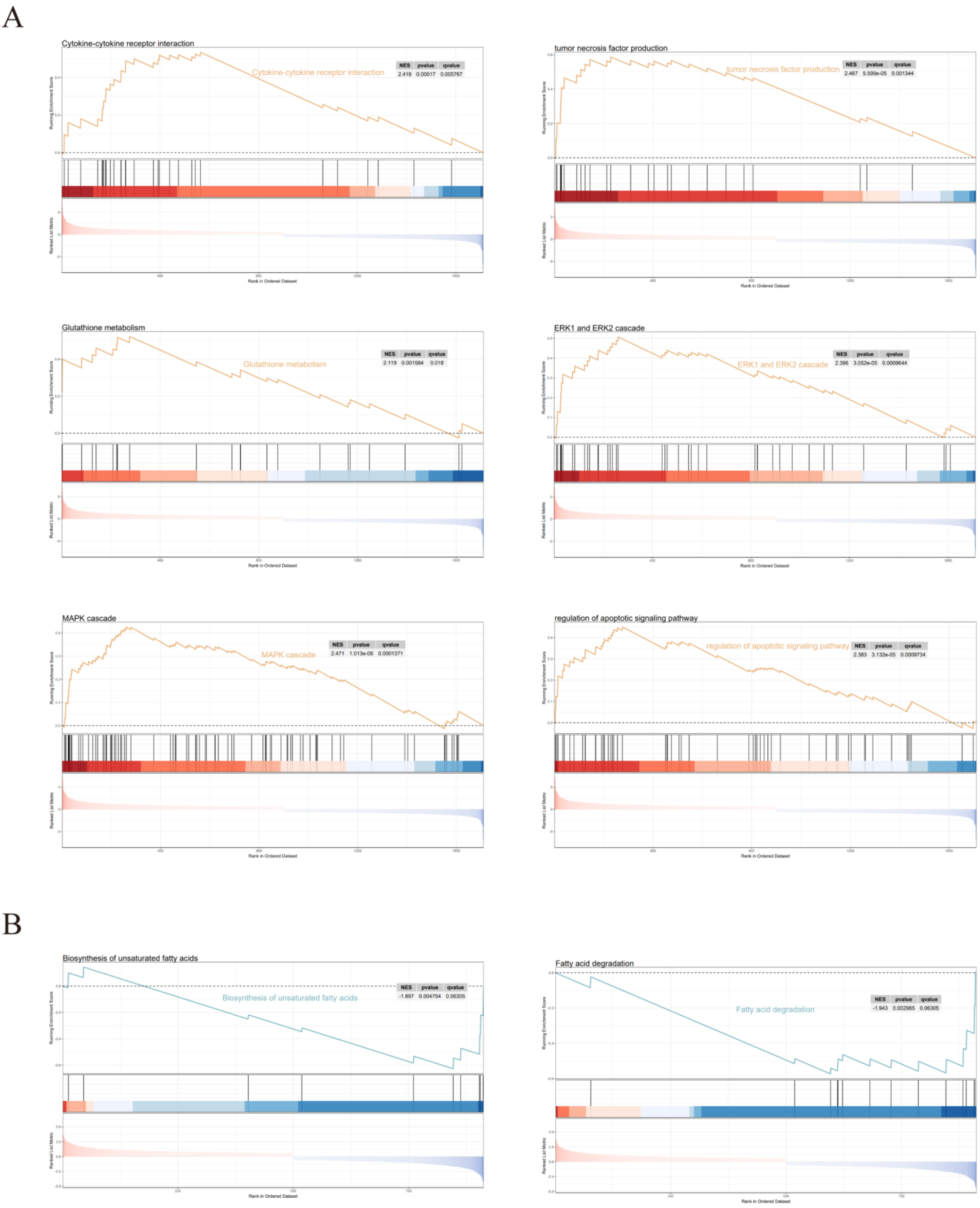

Figure S3. Gene set enrichment analysis (GSEA). GSEA of **(A)** APAP group vs. Ctrl group and **(B)** si-*Cyp2e1* LNPs treatment group vs. APAP group.

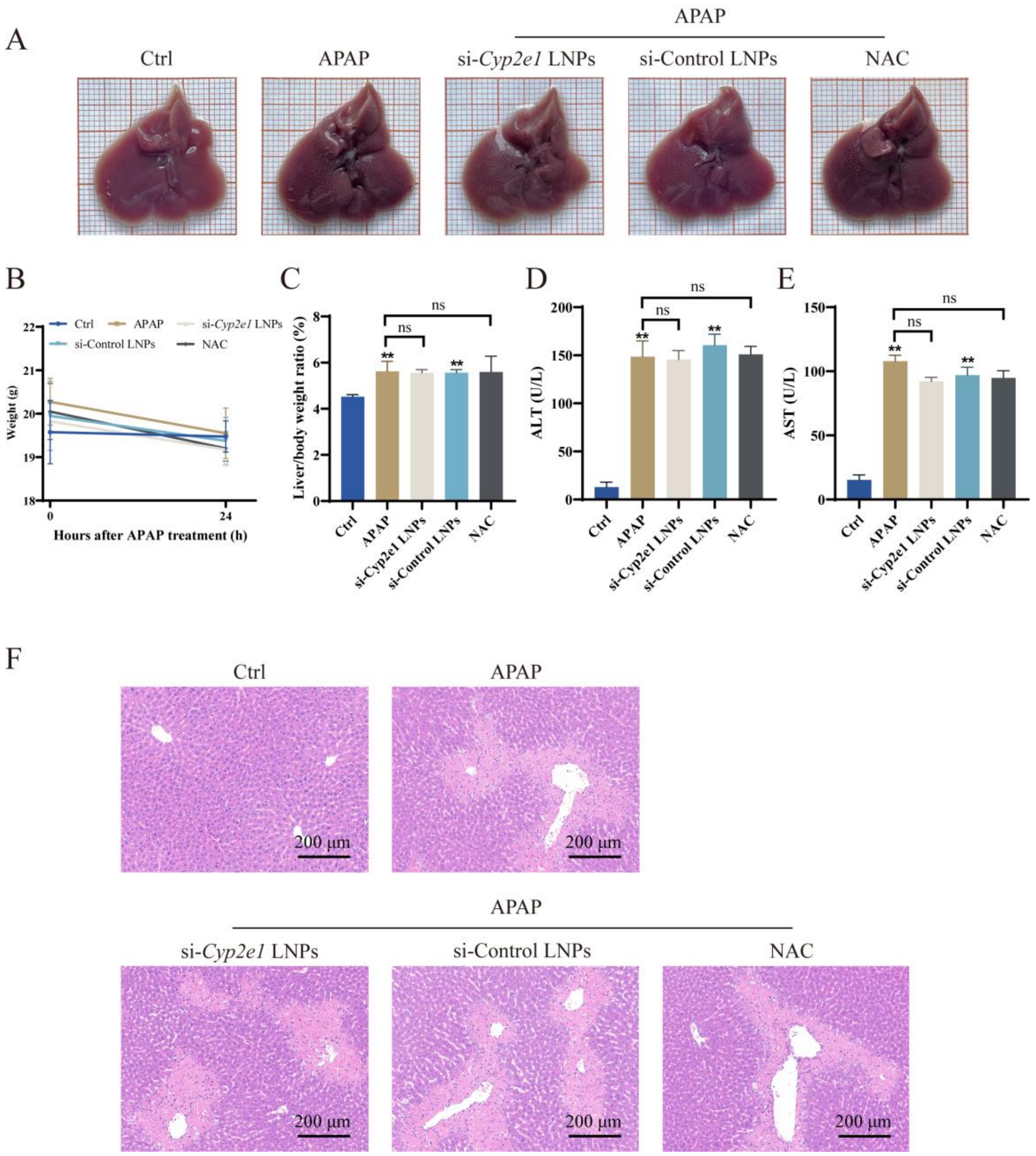

Figure S4. Effects of 2 h delay treatment with si-*Cyp2e1* LNPs on mice at 24 h after 300 mg/kg APAP injection (n = 4). **(A)** Appearance morphology of mouse liver. **(B)** Changes in body weight of mice. **(C–E)** Levels of liver index, serum ALT and AST. **(F)** Liver histology analysis of H&E staining (scale bars = 200 μm). ** $p < 0.01$ vs. Ctrl group.

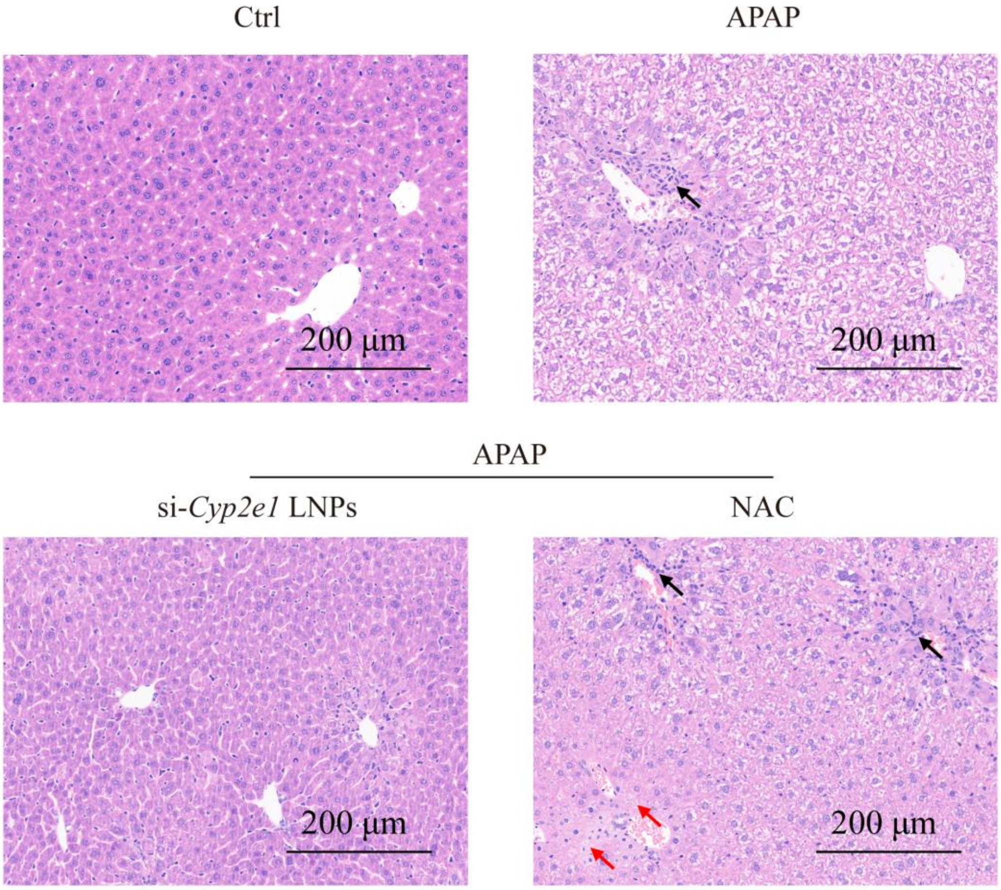

Figure S5. H&E staining of mouse liver 72 h after 300 mg/kg APAP injection (scale bars = 200 μm). The si-*Cyp2e1* LNPs treatment group received a single dose of 0.5 mg/kg si-*Cyp2e1* LNPs 2 h after APAP administration, while the NAC group were given a dose of 100 mg/kg NAC every 12 h.
