# Supplementary material for: Amelioration of Acetaminophen-Induced Hepatic Oxidative Stress and Inflammation by RNAi Targeting Cyp2e1 In Vivo
Source: Curr Issues Mol Biol. 2025 May 19;47(5):372. doi: 10.3390/cimb47050372 (PMC12110742; doi:10.3390/cimb47050372)
Supplement: Supplementary file 1 [file cimb-47-00372-s001.zip › cimb-3622004-supplementary/Supplementary Tables.pdf]

Table S1. Small interfering RNAs (siRNAs) sequences.

siRNA	Sequences (5′–3′)
si-*Cyp2e1* sense	ccAuGuAcAcAAuGGAAAAdTsdT [1]
si-*Cyp2e1* antisense	UUUUCcAUUGUGuAcAUGGdTsdT
si-Control sense	cuuAcGcuGAGuAcuucGAdTsdT
si-Control antisense	UCGAAGuACUcAGCGuAAGdTsdT

[1] The 2′-OMe modified nucleotides are in lower case, and the phosphorothioate linkages are indicated by "s".

Table S2. Primers of qPCR.

Gene	Primer	Sequences (5′–3′)
Cyp2e1	Forward	GCTGAGTACTCCCTGGATCC
	Reverse	CATGGGTTCTTGGCTGTGTT
Cyp4a10	Forward	TTCCCTGATGGACGCTCTTTA
	Reverse	GCAAACCTGGAAGGGTCAAAC
Cyp4a14	Forward	TTTAGCCCTACAAGGTACTTGGA
	Reverse	GCAGCCACTGCCTTCGTAA
Cd36	Forward	AGATGACGTGGCAAAGAACAG
	Reverse	CCTTGGCTAGATAACGAACTCTG
Fabp1	Forward	ATGAACTTCTCCGGCAAGTACC
	Reverse	CTGACACCCCCTTGATGTCC
Pparα	Forward	AACATCGAGTGTCGAATATGTGG
	Reverse	CCGAATAGTTCGCCGAAAGAA
Il-6	Forward	TAGTCCTTCCTACCCCAATTTCC
	Reverse	TTGGTCCTTAGCCACTCCTTC
Il-1β	Forward	GCAACTGTTCCTGAACTCAACT
	Reverse	ATCTTTTGGGGTCCGTCAACT
Tnf-α	Forward	CCCTCACACTCAGATCATCTTCT
	Reverse	GCTACGACGTGGGCTACAG
Gapdh	Forward	AGGTCGGTGTGAACGGATTTG
	Reverse	TGTAGACCATGTAGTTGAGGTCA
