# Supplementary figures and images for: Amelioration of Acetaminophen-Induced Hepatic Oxidative Stress and Inflammation by RNAi Targeting Cyp2e1 In Vivo
Source: Curr Issues Mol Biol. 2025 May 19;47(5):372. doi: 10.3390/cimb47050372 (PMC12110742; doi:10.3390/cimb47050372)

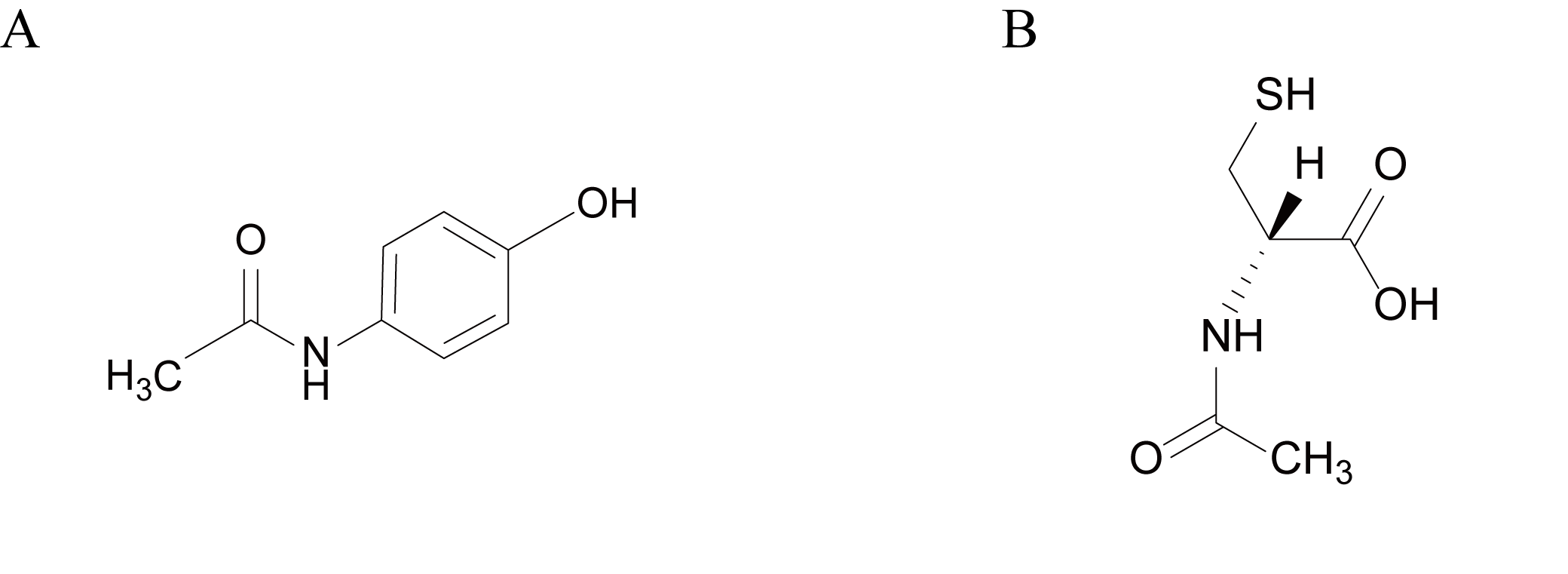

Supplement: Supplementary file 1 [file cimb-47-00372-s001.zip › cimb-3622004-supplementary/Supplementary Figures/Figure S1.tif]

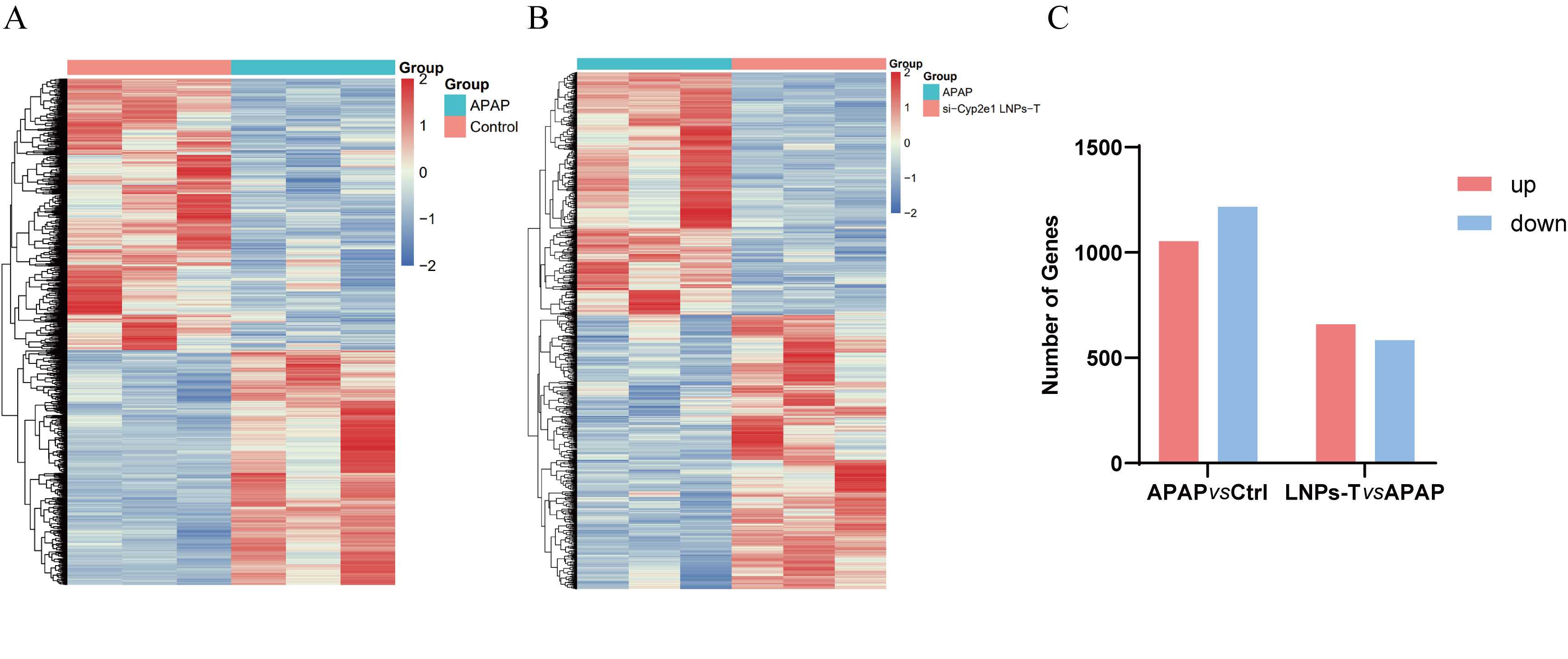

Supplement: Supplementary file 1 [file cimb-47-00372-s001.zip › cimb-3622004-supplementary/Supplementary Figures/Figure S2.tif]

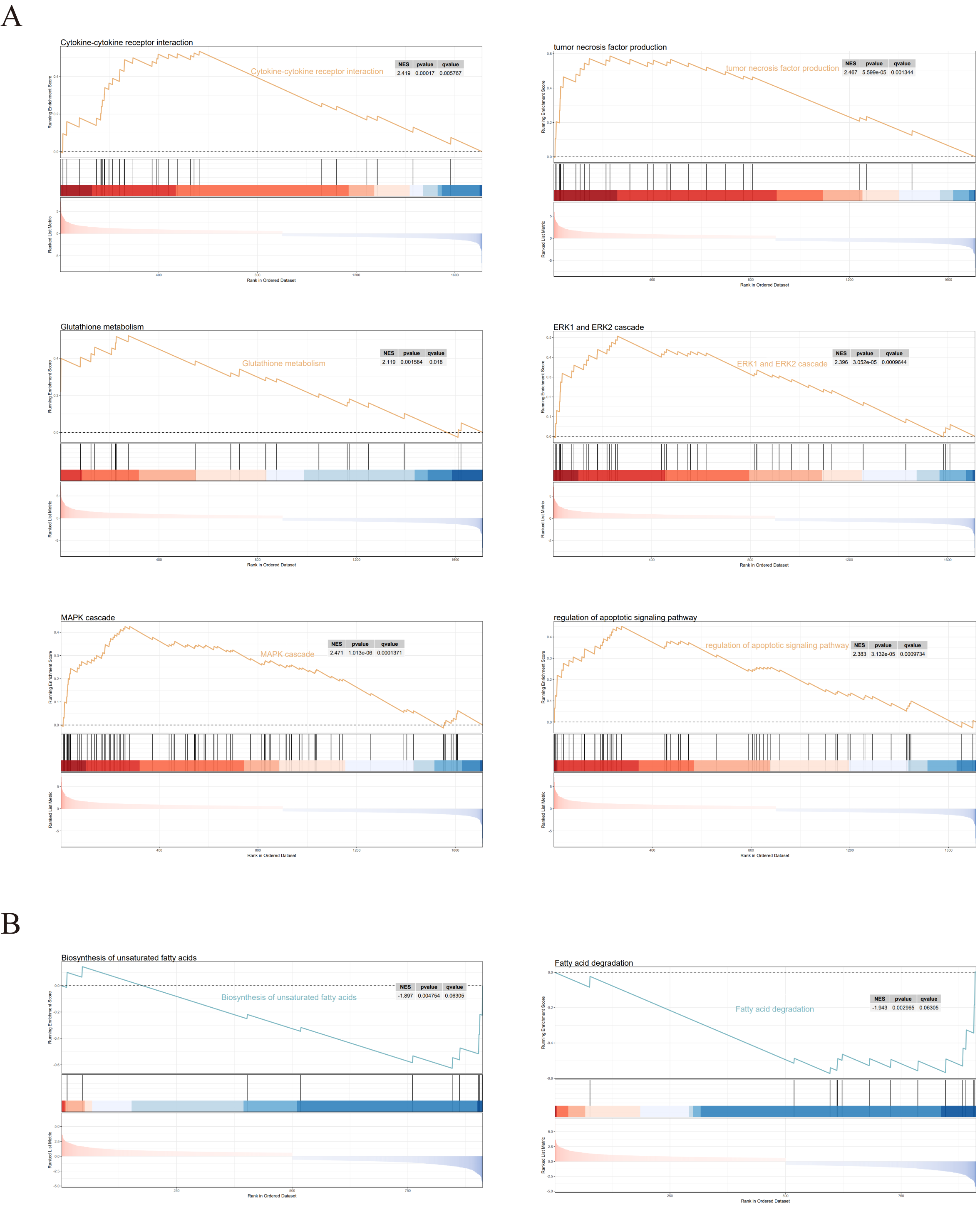

Supplement: Supplementary file 1 [file cimb-47-00372-s001.zip › cimb-3622004-supplementary/Supplementary Figures/Figure S3.tif]

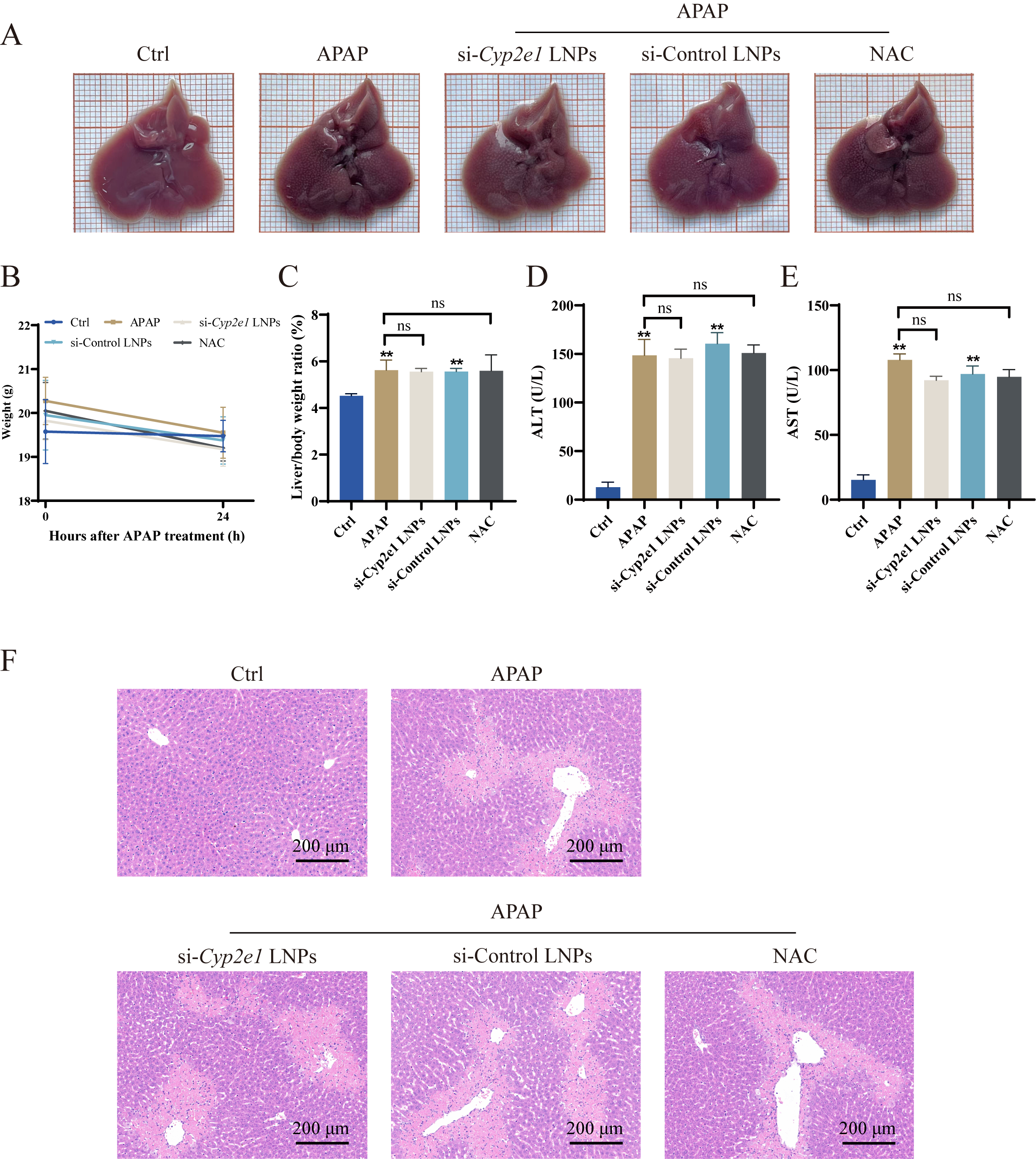

Supplement: Supplementary file 1 [file cimb-47-00372-s001.zip › cimb-3622004-supplementary/Supplementary Figures/Figure S4-MDPI.tif]

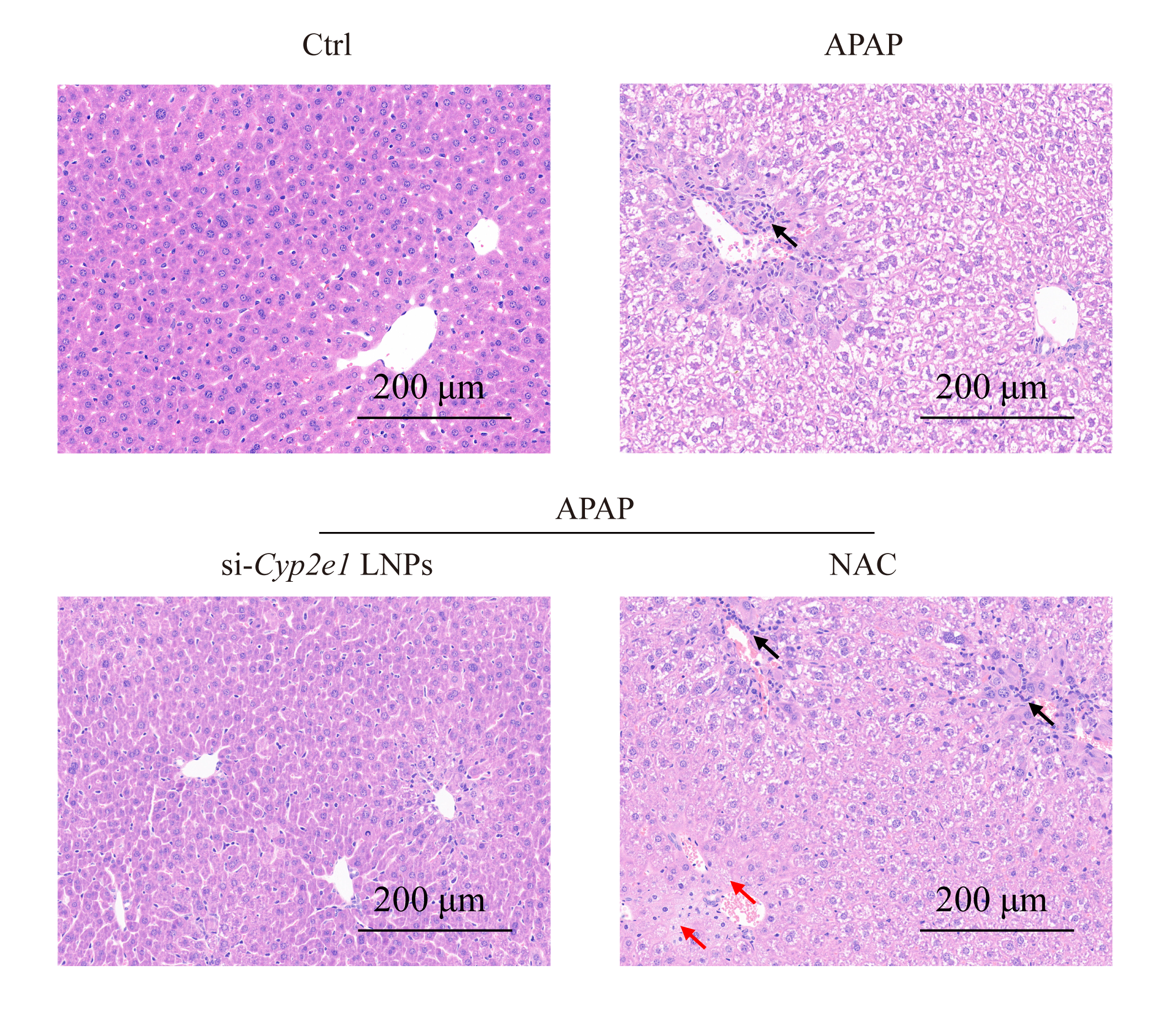

Supplement: Supplementary file 1 [file cimb-47-00372-s001.zip › cimb-3622004-supplementary/Supplementary Figures/Figure S5.tif]
